# Long Noncoding RNA RMRP Contributes to Paclitaxel Sensitivity of Ovarian Cancer by Regulating miR-580-3p/MICU1 Signaling

**DOI:** 10.1155/2022/8301941

**Published:** 2022-01-29

**Authors:** Lingling Li, Saitian Zeng, Liang Guo, Ping Huang, Jie Xi, Jing Feng, Qian Li, Yanying Li, Xiyun Xiao, Ruixue Yan, Jiyan Zhang

**Affiliations:** Department of Gynaecology, Cangzhou Central Hospital, Cangzhou, Hebei Province, China

## Abstract

Ovarian cancer is a prevalent female malignancy affecting the health and life of an increasing population of women around the world. Paclitaxel (PTX) resistance is a significant clinical problem in the treatment of ovarian cancer. However, the regulation mechanism of PTX resistance remains unclear. In this investigation, we reported an innovative function of the long noncoding RNA RMRP in promoting PTX resistance and glycolysis of ovarian cancer cells. We observed that RMRP was highly expressed in the ovarian cancer samples, in which the expression of RMRP was elevated in the PTX-resistant patients compared with the PTX-sensitive patients. Meanwhile, RMRP was upregulated in PTX-resistant ovarian cancer cell lines. Functionally, we found that the silencing of RMRP by siRNA significantly enhanced the PTX sensitivity of PTX-resistant ovarian cancer cells, in which the IC50 of PTX was reduced by RMRP depletion. The RMRP knockdown reduced cell viabilities and enhanced cell apoptosis of PTX-resistant ovarian cancer cells. Moreover, we observed that glucose uptake was enhanced in PTX-resistant ovarian cancer cells. The depletion of RMRP decreased glucose uptake, lactate product, and ATP production in PTX-resistant ovarian cancer cells. About the mechanism, we identified that RMRP was able to sponge miR-580-3p to enhance mitochondrial calcium uptake 1 (MICU1) expression in PTX-resistant ovarian cancer cells. MICU1 overexpression and miR-580-3p repression could reverse the RMRP-inhibited proliferation of PTX-resistant ovarian cancer cells in vitro. Thus, we concluded that RMRP contributes to PTX resistance and glycolysis of ovarian cancer by enhancing MICU1 expression through sponging miR-580-3p. Targeting RMRP may serve as a potential therapeutic strategy for the treatment of PTX-resistant ovarian cancer patients.

## 1. Introduction

Epithelial ovarian cancer ranks as the most lethal gynecological cancer, severely threatening the quality of life of women around the world [1]. The primary cause of the high lethality is mainly due to the lack of typical early symptoms and screening manners [2]. Hence, most patients with ovarian cancer are not diagnosed until an advanced stage [2]. The global standard of clinical management of ovarian cancer mainly includes surgical operations and chemotherapy [3]. Despite the fast development of therapeutic means, paclitaxel and carboplatin have remained the most prevalent chemotherapy agents for ovarian cancer for the past decades [3]. Nevertheless, tumor recurrence occurs following the long-term administration of chemotherapy drugs due to the development of multidrug resistance (MDR) [1, 3]. Among the developed drug resistance, paclitaxel (PTX) resistance constantly contributes to the failure of ovarian cancer clinical therapy [3]. Increasing evidence demonstrate that abnormal metabolic states, especially aerobic glycolysis, are involved in the chemoresistance of tumors [4]. However, the understanding of the specific mechanisms of glycolysis regulating ovarian cancer chemoresistance is still limited.

Long noncoding RNAs (lncRNAs) are transcripts with over 2000 nucleotides in sequence that do not translate into functional proteins [5]. LncRNAs work by regulating gene expression at both transcriptional and post-transcriptional levels, particularly functioning as sponges of miRNAs and interfering with their functions [6]. It is well recognized that lncRNAs are involved in various biological processes during carcinogenesis, profoundly affecting cancer development as tumor activators or suppressors [7, 8]. For example, lncRNA LINRIS promotes colorectal cancer progression via regulating MYC-associated glycolysis [8]. LncRNA RMRP is related to the growth and metastasis of multiple malignancies including hepatocellular carcinoma, bladder cancer, and nonsmall cell lung cancer [9, 10].

MiRNAs are a class of short noncoding RNAs with around 22 nucleotides in length, which are capable of suppressing mRNA translation to proteins via interacting with the 3′UTR of mRNA and causing degradation [11]. MiRNAs were also closely related to the development of intrinsic or acquired drug resistance [12]. For instance, Au Yeung et al. suggested that miR21 delivered by exosomes facilitated paclitaxel resistance of ovarian cancer cells [13]. Previous studies reported miR-580 as a negative regulator of TWIST expression in breast cancer [14], suggesting the potential role of miR-580-2p in tumor progression.

Mitochondrial calcium uptake 1 (MICU1) is a critical regulator that controls Ca^2+^ uptake in mitochondria, mediates oxidative metabolism, and helps avoid accumulation of mitochondrial Ca^2+^ and cell death [15–17]. A recent study demonstrated that MICU1 was able to activate glycolysis and contributed to cisplatin-resistance in ovarian cancer, consequently promoting tumor growth and metastasis [18], suggesting its potential role in PTX-resistant ovary cancer. However, very limited studies have detailed the regulatory mechanisms of MICU1, especially the participation of miRNAs in MICU1-regulated tumor progression [19].

In this study, we found an elevated level of RMRP in ovary cancer patients that were resistant to PTX therapy, and the depletion of RMRP could notably suppress glycolysis. Further study on molecular mechanisms presented the interaction of RMRP with miR-580-3p and identified MICU1 as the target of miR-580-3p. This work presented lncRNA RMRP as a potential promoter of glycolysis and chemoresistance in ovary cancer, hinting at a potential target for adjuvant treatment of ovary cancer with PTX resistance.

## 2. Materials and Methods

### 2.1. Patient Samples

The tumor samples and corresponding adjacent normal tissues were collected from 36 patients with ovarian cancer hospitalized at Cangzhou Central Hospital. The study was authorized by the ethics committee of Cangzhou Central Hospital. All patients were informed of the study and signed informed consent. Patient information was recorded, and 18 of the enrolled patients showed PTX resistance, which was defined by tumor recurrence within 6 months after standard chemotherapy.

### 2.2. Cell Culture and Treatment

Ovarian cancer cell lines SKOV3 and HeyA-8 were purchased from the Chinese Academy of Sciences Cell Bank of Type Culture Collection. The culturing of cells was carried out in a complete DMEM/F12 medium (Gibco, USA) added with 100 U/mL penicillin and 100 mg/L streptomycin (Sigma, USA). To achieve PTX-resistant, SKOV3 and HeyA-8 cells were constantly placed in culturing medium containing a middle-lethal dose of PTX (Sigma) and maintained in a culture medium with 10 nM PTX. The acquired PTX-resistant cell lines were named SKOV3/PTX and HeyA-8/PTX, respectively. All cells were incubated in a 37°C incubator filled with a humidified atmosphere containing 5% CO_2_.

### 2.3. Quantitative Real-Time PCR

Total RNA was extracted from patient samples and cell lines of ovarian cancer by using a TRIzol solution (Sigma) following the manufacturer's protocol. The RNAs were quantified. A total amount of 1 *μ*g RNA was reverse transcribed to cDNA by a cDNA Reverse Transcription Kit (Beyotime, China). A quantitative real-time PCR experiment was processed using a Talent qPCR PreMix (TIANGEN, China) according to the manufacturer's instructions, and the values were detected by an Applied Biosystem (Thermo, USA). The relative levels of RMRP, miR-580-3p, and MICU1 were calculated by using a 2^−ΔΔCt^ method. The *β*-actin and U6 were used as internal control for normalization of mRNA and miRNA RNAs, respectively.

### 2.4. Cell Transfection

MiR-580-3p mimics, RMRP shRNA (shRMRP), and their corresponding negative control sequences were purchased from Qiagen (China). Transfection of miR-580-3p mimics and shRMRP into SKOV3/PTX and HeyA-8/PTX were processed by Lipofectamine 3000 reagent (Invitrogen, USA) under the instructions of the manufacturer. The efficacy of miR-580-3p mimics and shRMRP were evaluated through a real-time PCR experiment.

### 2.5. Cell Viability

The cell viability was assessed by a 3-(4, 5- dimethylthiazol-2-yl)-2, 5-diphenyltetrazolium bromide (MTT) experiment. SKOV3/PTX, HeyA-8/PTX, and their parental cells were treated as indicated in each experiment, digested, suspended as single cells, and planted in 96-well plates (3 × 10^3^ cells per well). At the end time point (24 h, 48 h, and 72 h), MTT reagent was added at a final concentration of 5 mg/ml in each well. Following a 4-hour incubation, the cell medium was discarded and replaced by 150 *μ*l of DMSO in each well. The plates were gently shaken in the dark for 10 minutes. Absorbance at 490 nm was detected.

### 2.6. Detection of Glycolysis

The biomarkers of glycolysis include glucose uptake, lactate products, and ATP production in cells. In this study, the levels of glucose uptake, lactate, and ATP in SKOV3/PTX, HeyA-8/PTX, and their parental cell lines, as well as cells after siRMRP transfection, were detected by using corresponding kits following the manufacturer's protocols. The Glucose Uptake Kit, Lactate Assay Kit, and Enhanced ATP Assay Kit were purchased from Beyotime. All experiments were performed in three replicates.

### 2.7. Luciferase Reporter Assay

The potential binding sites between miR-580-3p with RMRP or the 3′UTR region of MICU1 were analyzed on the TargetScan website (http://www.targetscan.org/vert_71/). The wild type sequences of RMRP and MICU1 were downloaded from the PubMed website, cloned, and inserted into the pMIR-REPORT plasmid (Promega, USA). RMRP-Mut and MICU1-Mut plasmids were obtained by mutating the sequences of the binding sites. RMRP-Mut and MICU1-Mut or their corresponding controls RMRP-WT and MICU1-WT were cotransfected with siRMRP or miR-580-3p mimics into 293T cells. After transfection for 24 hours, the luciferase activity was examined by a dual-luciferase reporter assay kit under the manufacturer's instructions.

### 2.8. RNA Pull-Down Assay

Biotin-labeled wild type or mutated miR-580-3p (GenePharma, China) were transfected into cells for 24 hours. After that, the cells were lysed by NP40 lysis buffer and incubated with Dynabeads (Invitrogen) for 3 hours in gentle rotation. The beads coupled with RNA were then collected and lysed by TRIzol reagent. The enrichment of RMRP was detected by qPCR.

### 2.9. Western Blotting

Cells were harvested and lysed on ice by the ice-cold RIPA cell lysis buffer, which was added with a 1× protease inhibitor cocktail (Beyotime). Protein concentration was evaluated by a BCA quantification method. Subsequently, a total amount of 35 µg of extracted proteins were separated on 10% SDS-PAGE and transferred onto NC membranes. The blots were probed with specific primary antibodies (anti-MICU1, 1 : 1000, Abcam, USA; *β*-actin, 1 : 2000, Abcam) at 4 C overnight, followed by incubation with secondary antibodies at room temperature for 45 minutes. The blots were washed with TBST three times, followed by visualization with a Millipore ECL reagent (Millipore, USA) under a gel imaging system (BD Biosciences, USA).

### 2.10. Flow Cytometry

The portion of apoptotic cells of SKOV3/PTX and HeyA-8/PTX cells after indicated transfection was measured by flow cytometry. Cells were seeded in 6-well plates and subjected to the aforementioned treatment, collected and stained with PI and FITC-Annexin V for 15 minutes, then the fluorescence intensity was examined by flow cytometry (BD Biosciences) immediately.

### 2.11. Statistical Analysis

The statistical analysis of the data in the present study was performed via the SPSS software (Version 19.0). Data were shown as mean ± SD. A two-sided Student's *t*-test was applied for comparison between two experimental groups, while the one-way ANOVA method was applied to compare the difference between multiple groups. *P* < 0.05 were set as the threshold for statistical significance.

## 3. Results

### 3.1. RMRP Is Highly Expressed in Ovarian Cancer Tissues and PTX-Resistant Cells

**Figure 1:**
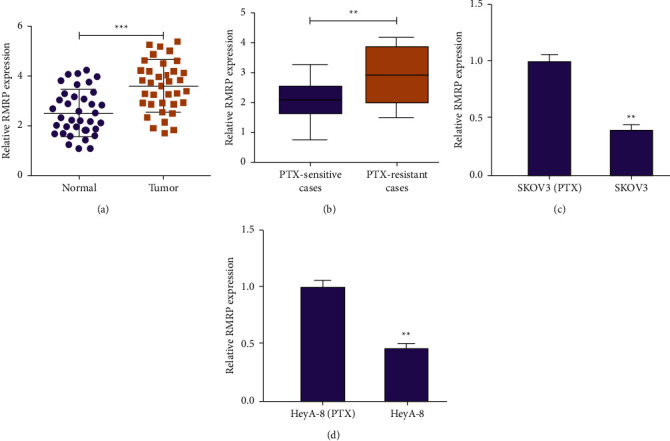
RMRP is highly expressed in ovarian cancer tissues and PTX-resistant cells. (a) The RMRP expression was analyzed in ovarian cancer patient tissues (*n* = 36) and the normal cases (*n* = 36) by qPCR. (b) The RMRP expression was detected in PTX-resistant (*n* = 16) and PTX sensitive (*n* = 16) ovarian cancer patient tissues by qPCR. (c d) The RMRP expression was measured in HeyA-8 and SKOV3 cells and PTX-resistant HeyA-8 (HeyA-8/PTX) and SKOV3 (SKOV3/PTX) cells by qPCR. Mean ± SD, ^*∗∗*^*P* < 0.01.

Firstly, we evaluated the expression of RMRP in clinical ovarian cancer samples. We found that RMRP was highly expressed in ovarian cancer patients (*n* = 36) compared with that of normal cases (*n* = 36) (Figure 1(a)). Specifically, RMRP expression was enhanced in PTX-resistant patients (*n* = 18) than in PTX-sensitive patients (*n* = 18) (Figure 1(b)). Moreover, the RMRP was elevated in PTX-resistant HeyA-8 (HeyA-8/PTX) and SKOV3 (SKOV3/PTX) cells (Figures 1(c) and 1(d)).

### 3.2. RMRP Contributes to the PTX Resistance of Ovarian Cancer Cells

**Figure 2:**
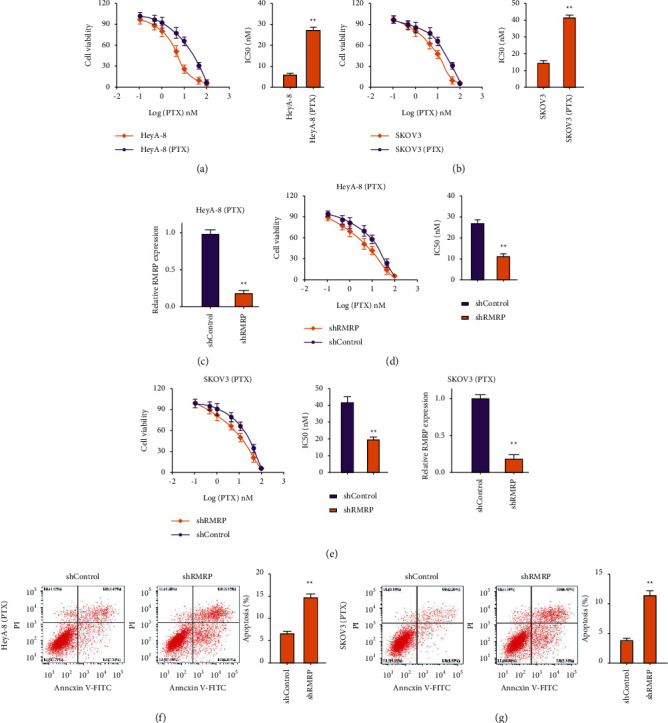
RMRP contributes to the PTX resistance of ovarian cancer cells. (a, b) The cell viabilities of HeyA-8 and SKOV3 cells and PTX-resistant HeyA-8 (HeyA-8/PTX) and SKOV3 (SKOV3/PTX) cells were detected by MTT assays. (c) The expression of RMRP was measured in HeyA-8/PTX and SKOV3/PTX cells by qPCR. (d–g) The HeyA-8/PTX and SKOV3/PTX cells were treated with RMRP shRNA. (d, e) The cell viabilities were analyzed by MTT assays in HeyA-8/PTX and SKOV3/PTX cells. (f, g) The cell apoptosis was detected by flow cytometry in HeyA-8/PTX and SKOV3/PTX cells. shcontrol, control shRNA; shRMRP, RMRP shRNA. Mean ± SD, ^*∗∗*^*P* < 0.01.

We then observed that HeyA-8/PTX and SKOV3/PTX cell lines presented an increased PTX IC50 compared with HeyA-8 and SKOV3 cell lines (Figures 2(a) and 2(b)). The expression of RMRP was silenced by siRNA in HeyA-8/PTX and SKOV3/PTX cells (Figure 2(c)). The depletion of RMRP by siRNA reduced the IC50 values of PTX in inhibiting HeyA-8/PTX and SKOV3/PTX cell viabilities (Figures 2(d) and 2(e)). Meanwhile, the knockdown of RMRP by siRNA enhanced HeyA-8/PTX and SKOV3/PTX cell apoptosis (Figures 2(f) and 2(g)).

### 3.3. RMRP Enhances Glycolysis of PTX-Resistant Ovarian Cancer Cells

**Figure 3:**
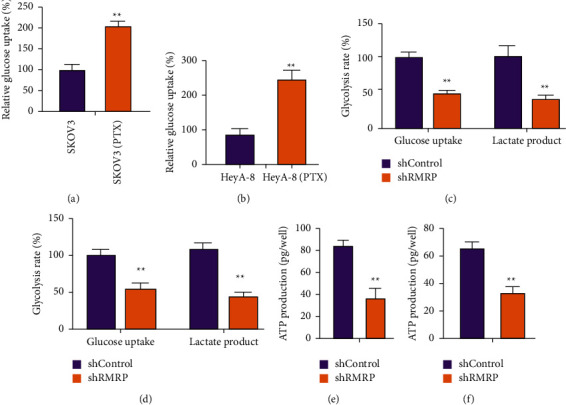
RMRP enhances glycolysis of PTX-resistant ovarian cancer cells. (a, b) The glucose uptake was measured in HeyA-8 and SKOV3 cells and PTX-resistant HeyA-8/PTX and SKOV3/PTX cells. (c–f) The HeyA-8/PTX and SKOV3/PTX cells were treated with RMRP shRNA. (c, d) The glucose uptake and lactate product were measured in HeyA-8/PTX and SKOV3/PTX cells. (e, f) The ATP production was analyzed in HeyA-8/PTX and SKOV3/PTX cells. shcontrol, control shRNA; shRMRP, RMRP shRNA. Mean ± SD, ^*∗∗*^*P* < 0.01.

We then assessed the correlation of PTX resistance with glycolysis in the HeyA-8/PTX and SKOV3/PTX cells. We found that the glucose uptake was enhanced in HeyA-8/PTX and SKOV3/PTX cells relative to HeyA-8 and SKOV3 cells (Figures 3(a) and 3(b)). The depletion of RMRP significantly reduced glucose uptake, lactate product, and ATP production in HeyA-8/PTX and SKOV3/PTX cells (Figures 3(c)–3(f)).

### 3.4. RMRP Is Able to Sponge miR-580-3p

**Figure 4:**
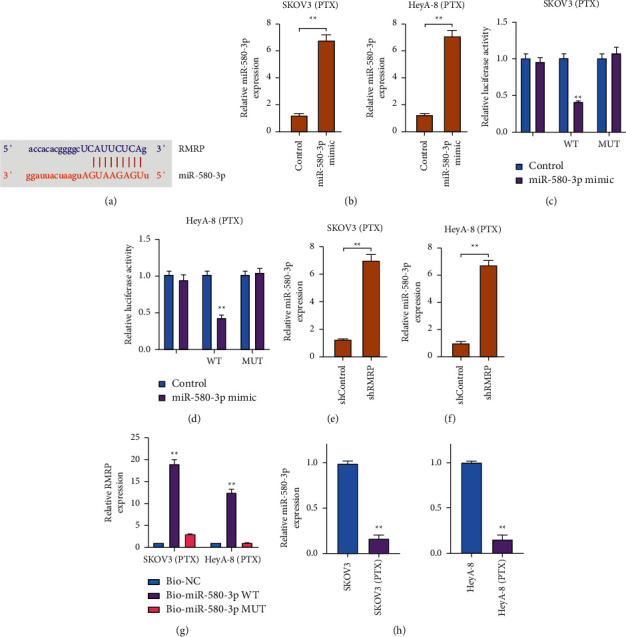
RMRP is able to sponge miR-580-3p. (a) The binding site prediction of RMRP and miR-580-3p in ENCORI database. (b–d) The HeyA-8/PTX and SKOV3/PTX cells were treated with miR-580-3p mimic. (b) The miR-580-3p expression was analyzed by qPCR. (c, d) The luciferase activity was detected by dual luciferase reporter assays. (e, f) The HeyA-8/PTX and SKOV3/PTX cells were treated with RMRP siRNA. The miR-580-3p expression was analyzed by qPCR. (g) The direct interaction of RMRP and miR-580-3p was analyzed by RNA pull down. (h) The miR-580-3p expression was measured in HeyA-8 and SKOV3 cells and PTX-resistant HeyA-8 (HeyA-8/PTX) and SKOV3 (SKOV3/PTX) cells by qPCR. shcontrol, control shRNA; shRMRP, RMRP shRNA. Mean ± SD, ^*∗∗*^*P* < 0.01.

To determine the mechanisms of RMRP-mediated PTX resistance in HeyA-8/PTX and SKOV3/PTX cells, the binding prediction analysis was performed in the ENCORI database. We found the potential interaction of RMRP with miR-580-3p (Figure 4(a)). The expression of miR-580-3p was enhanced by miR-580-3p mimic in HeyA-8/PTX and SKOV3/PTX cells (Figure 4(b)). The miR-580-3p mimic reduced the luciferase activity of RMRP in HeyA-8/PTX and SKOV3/PTX cells (Figures 4(c) and 4(d)). The silencing of RMRP repressed miR-580-3p expression in HeyA-8/PTX and SKOV3/PTX cells (Figures 4(e) and 4(f)). The direct interaction of RMRP and miR-580-3p was validated by RNA pull down in cells (Figure 4(g)). Moreover, the miR-580-3p was reduced in PTX-resistant HeyA-8/PTX and SKOV3/PTX cells (Figure 4(h)). Meanwhile, miR-580-3p reduced the IC50 values of PTX in inhibiting HeyA-8/PTX and SKOV3/PTX cell viabilities (Figures S1A and B). The treatment of miR-580-3p mimics enhanced HeyA-8/PTX and SKOV3/PTX cell apoptosis (Figures S1C and D).

### 3.5. MiR-580-3p Is Able to Target MICU1 in Ovarian Cancer Cells

**Figure 5:**
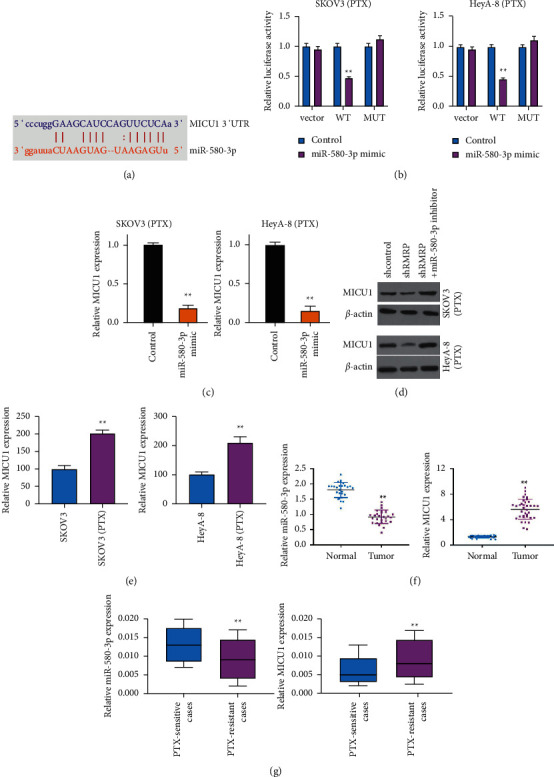
MiR-580-3p is able to target MICU1 in ovarian cancer cells. (a) The binding site prediction of MICU1 and miR-580-3p in ENCORI database. (b, c) The HeyA-8/PTX and SKOV3/PTX cells were treated with miR-580-3p mimic. (b) The luciferase activity was detected by dual luciferase reporter assays. (c) The expression of MICU1 was detected by qPCR. (d) The HeyA-8/PTX and SKOV3/PTX cells were co-treated with RMRP shRNA and miR-580-3p inhibitor. The expression of MICU1 was detected by qPCR. (e) The MICU1 expression was measured in HeyA-8 and SKOV3 cells and PTX-resistant HeyA-8 (HeyA-8/PTX) and SKOV3 (SKOV3/PTX) cells by qPCR. (f) The MICU1 and miR-580-3p expression was analyzed in ovarian cancer patient tissues (*n* = 36) and the normal cases (*n* = 36) by qPCR. (g) The MICU1 and miR-580-3p expression was detected in PTX-resistant (*n* = 16) and PTX sensitive (*n* = 16) ovarian cancer patient tissues by qPCR. Mean ± SD, ^*∗∗*^*P* < 0.01.

We then identified the binding sites of miR-580-3p and MICU1 (Figure 5(a)). The luciferase activity and mRNA expression of MICU1 were repressed by miR-580-3p mimic in HeyA-8/PTX and SKOV3/PTX cells (Figures 5(b) and 5(c)). The knockdown of RMRP-inhibited MICU1 expression while miR-580-3p reversed the inhibition of MICU1 expression in HeyA-8/PTX and SKOV3/PTX cells (Figure 5(d)). The MICU1 was enhanced in PTX-resistant HeyA-8/PTX and SKOV3/PTX cells (Figure 5(e)). MICU1 was highly expressed, and miR-580-3p was reduced in ovarian cancer patients (*n* = 36) compared with normal cases (*n* = 36) (Figure 5(f)). Specifically, the MICU1 expression was enhanced and miR-580-3p expression was inhibited in PTX-resistant patients (*n* = 18) compared to PTX-sensitive patients (*n* = 18) (Figure 5(g)).

**Figure 6:**
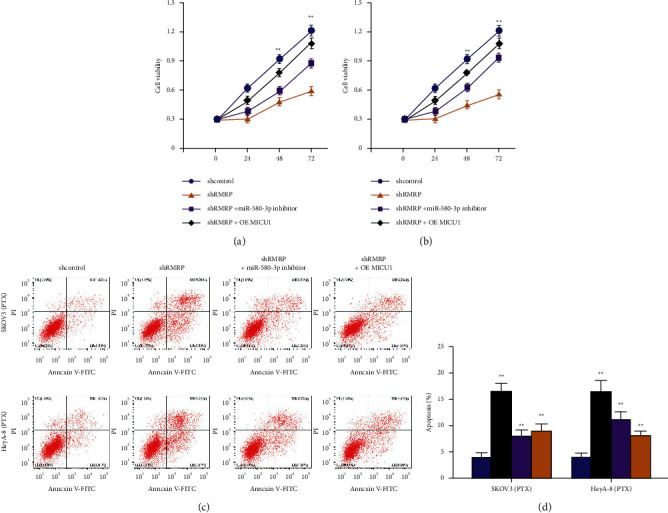
RMRP/miR-580-3p/MICU1 axis regulates progression of ovarian cancer cells. (a–d) The HeyA-8/PTX and SKOV3/PTX cells were co-treated with RMRP siRNA and miR-580-3p inhibitor or pcDNA-MICU1 overexpression plasmid. (a, b) The cell viabilities of were analyzed by MTT assays. (c, d) The cell apoptosis was detected by flow cytometry. shcontrol, control shRNA; shRMRP, RMRP shRNA; OE MICU1, MICU1 overexpression. Mean ± SD, ^*∗∗*^*P* < 0.01.

Next, we found that the depletion of RMRP was able to inhibit cell proliferation and induce cell apoptosis while the inhibition of miR-580-3p or the overexpression of MICU1 could reverse this effect in HeyA-8/PTX and SKOV3/PTX cells (Figure 6).

## 4. Discussion

Ovarian cancer is a prevalent female malignancy with high recurrence, and PTX resistance is a significant clinical problem. The mechanism underlying the regulation of PTX resistance of ovarian cancer cells remains obscure. In this study, we provide new evidence of lncRNA RMRP contributing to PTX resistance of ovarian cancer cells.

It has been reported that RMRP is widely involved in the modulation of cancer development. LncRNA RMRP contributes to invasion, migration, and proliferation by targeting miR-206 in bladder cancer [10]. LncRNA RMRP enhances invasion, migration, and proliferation of nonsmall cell lung cancer cells by the miR-613/NFAT5 axis [20]. LncRNA RMRP inhibition represses malignant progression of hepatocellular carcinoma by regulating miRNA-206/TACR1 signaling [21]. These studies indicate that RMRP plays a crucial role in cancer progression. Meanwhile, several lncRNAs have been identified in regulating PTX resistance of ovarian cancer. LncRNA FER1L4 reduces PTX resistance of ovarian cancer cells by targeting MAPK signaling [22]. LncRNA TUG1 contributes to autophagy-modulated PTX resistance of ovarian cancer cells by repressing miR-29b-3p [23]. LncRNA PRLB promotes PTX resistance by targeting NF-*κ*B signaling in ovarian cancer cells [24]. These reports suggest that lncRNAs are involved in the modulation of PTX resistance in ovarian cancer. Importantly, the correlation of PTX resistance and glycolysis in cancer has been reported [25]. Our data further showed that RMRP contributes to PTX resistance and glycolysis in ovarian cancer cells. It indicates a critical function of RMRP in regulating ovarian cancer development, especially PTX resistance and glycolysis. Our data provide valuable evidence of the crucial roles of lncRNAs in *e* PTX resistance of ovarian cancer. The significance of RMRP in chemotherapy resistance in other cancers may need to be explore in the future.

Moreover, it has been reported that MICU1 contributes to chemoresistance and glycolysis of ovarian cancer cells [18]. MicroRNA-195 targets MICU1 expression to regulate ovarian cancer cell growth [16]. Besides, circular RNA hsa_circ_0072309 represses the progression of nonsmall cell lung cancer progression via targeting miR-580-3p [26]. A LHFPL3-AS1/miR-580-3p/STAT3 axis contributes to melanoma development by activating the JAK2/STAT3 pathway [27]. The function of miR-580-3p in ovarian cancer remains unclear. In our investigation, RMRP was able to enhance MICU1 expression by sponging miR-580-3p in ovarian cancer cells. MICU1 and miR-580-3p were involved in the RMRP-mediated proliferation of PTX-resistant ovarian cancer cells. These data present one potential mechanism of RMRP-mediated PTX resistance and cancer progression. RMRP-regulated PTX resistance and progression of ovarian cancer cells at least partly by targeting the miR-580-3p/MICU1 axis. Our finding provides new insights into the mechanism by which RMRP regulates PTX resistance and glycolysis of ovarian cancer by modulating the miR-580-3p/MICU1 axis. Meanwhile, the miR-580-3p/MICU1 axis may just be one of the mechanisms of RMRP-regulated ovarian cancer and other downstream factors should be explored in future investigations.

Thus, we concluded that RMRP contributes to PTX resistance and glycolysis of ovarian cancer by enhancing MICU1 expression through sponging miR-580-3p. Targeting RMRP may serve as a potential therapeutic strategy for the treatment of PTX-resistant ovarian cancer patients.

## Data Availability

The datasets used and analyzed during the current study are available from the corresponding author on reasonable request.

## Supplementary Materials

Supplementary Materials Figure S1: MiR-580-3p represses PTX resistance of ovarian cancer cells. (A–D) The HeyA-8/PTX and SKOV3/PTX cells were treated with miR-580-3p mimics. (A and B) The cell viabilities were analyzed by MTT assays in HeyA-8/PTX and SKOV3/PTX cells. (C and D) The cell apoptosis was detected by flow cytometry in HeyA-8/PTX and SKOV3/PTX cells. mean ± SD, ^*∗∗*^*P* < 0.01.
