# Supplementary material for: Long Noncoding RNA RMRP Contributes to Paclitaxel Sensitivity of Ovarian Cancer by Regulating miR-580-3p/MICU1 Signaling
Source: J Oncol. 2022 Jan 29;2022:8301941. doi: 10.1155/2022/8301941 (PMC8817877; doi:10.1155/2022/8301941)
Supplement: Supplementary Materials — Figure S1: MiR-580-3p represses PTX resistance of ovarian cancer cells. (A–D) The HeyA-8/PTX and SKOV3/PTX cells were treated with miR-580-3p mimics. (A and B) The cell viabilities were analyzed by MTT assays in HeyA-8/PTX and SKOV3/PTX cells. (C and D) The cell apoptosis was detected by flow cytometry in HeyA-8/PTX and SKOV3/PTX cells. mean ± SD, ∗∗P < 0.01. [file 8301941.f1.docx]

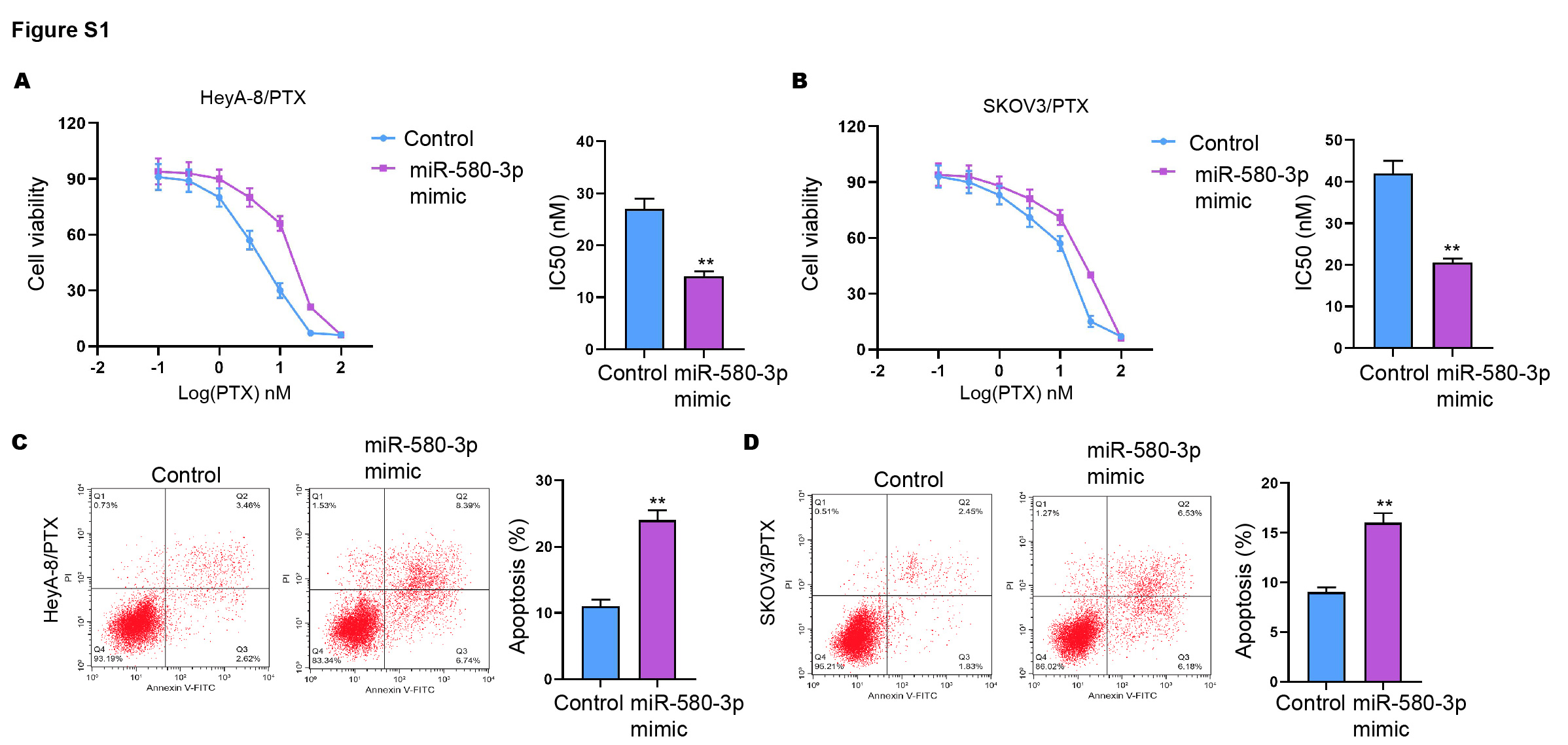


**Fig. S1. MiR-580-3p represses PTX resistance of ovarian cancer cells.** (A-D) The HeyA-8/PTX and SKOV3/PTX cells were treated with miR-580-3p mimics. (A and B) The cell viabilities were analyzed by MTT assays in HeyA-8/PTX and SKOV3/PTX cells. (C and D) The cell apoptosis was detected by flow cytometry in HeyA-8/PTX and SKOV3/PTX cells. mean ± SD, ** *P* < 0.01.
